# TB-Related Stigma: A Hidden Obstacle to Adherence Monitoring with Video Directly Observed Treatment Among Patients with Tuberculosis in Uganda

**DOI:** 10.21203/rs.3.rs-7447805/v1

**Published:** 2025-09-01

**Authors:** Kelvin Bwambale, Damalie Nakkonde, Gloria Nassanga, Esther Buregyeya, Sarah Zalwango, Juliet N. Sekandi

**Affiliations:** Makerere University; Makerere University; Makerere University; Makerere University; Kampala Capital City Authority; University of Georgia

**Keywords:** Video Directly Observed Therapy (VDOT), Tuberculosis, TB-related stigma, Self-stigma, Anticipated stigma, Public-stigma, Uganda

## Abstract

**Background:**

Adherence to video directly observed treatment (VDOT) remains inconsistent, with some patients frequently missing video submissions. Stigma associated with tuberculosis (TB) may influence patients’ engagement with VDOT, leading to non-adherence. This study examines the effect of baseline TB-related stigma on missed VDOT submissions as a marker of patient engagement throughout treatment among patients with TB.

**Methods:**

This study was a secondary analysis of 71 patients with TB from the DOT Selfie Randomized clinical trial (RCT) in Kampala, Uganda (July 2020–October 2021). It focused on the association between baseline TB-related stigma and missed video submissions during six months of VDOT. Stigma was measured using a 13-item tool covering self-, anticipated, and public stigma. Self-stigma is when individuals believe and internalize negative views about TB, causing shame and avoiding care. Public stigma involves harmful attitudes and discrimination from others, leading to isolation. Anticipated stigma is the fear of being judged or treated unfairly if one’s TB status becomes known. Four negative binomial regression models adjusted for HIV status, alcohol use, household size, marital status, and TB severity were used to estimate adjusted incidence rate ratios (aIRR) with their 95% confidence intervals (CIs) using STATA 14.2.

**Results:**

The study included 71 patients with TB with a mean age of 33 years (SD = 12), and 36 (51%) were female. TB-related stigma was highly prevalent, with 51% (95% CI: 39–62) experiencing high levels of overall stigma. The prevalence of public stigma was 97% (95% CI: 90–100), self-stigma was 80% (95% CI: 22–45), and anticipated stigma was 68% (95% CI: 55–78). High overall stigma was significantly associated with an increase in the rate of missed VDOT videos (aIRR = 1.9; 95% CI: 1.1–3.5). Similarly, patients who reported anticipated stigma missed twice as many VDOT videos as those without anticipated stigma (aIRR: 2.1; 95% CI: 1.2–3.8). There was no significant association between self and public stigma and missed videos.

**Conclusion:**

TB-related stigma, particularly the anticipated fear of judgment, undermines VDOT adherence monitoring. Interventions such as early screening for stigma, patient counselling, and community education are essential to improving outcomes.

## Background

Tuberculosis (TB) reclaimed its position as the leading cause of death from a single infectious disease in 2023, surpassing COVID-19 after three years^1^. An estimated 10.8 million people developed TB (twice the population of Arizona state), and 1.25 million TB-related deaths were recorded globally^1^. The burden remains disproportionately high in low- and middle-income countries, with the Southeast Asia and African WHO regions accounting for 45% and 24% of global TB cases, respectively^2^. Poor adherence to TB treatment is one of the critical barriers to achieving these targets.^3,4^. Studies indicate that treatment non-adherence, including both treatment interruptions and default, ranges from 8% to 60% globally, with higher rates observed in resource-limited settings^3,5,6^. Consequently, TB treatment non-adherence increases infectiousness, risk of multidrug-resistant TB (MDR-TB), and poor treatment outcomes, including higher mortality rates^7–11^.

While directly observed treatment (DOT) has been effective in improving adherence, its implementation is limited by significant logistical and resource constraints. Digital adherence technologies (DATs), particularly VDOT, have emerged as innovative solutions for remotely monitoring medication adherence^12,13^. VDOT enables patients with TB to record and submit videos of themselves taking medication via a smartphone application, allowing healthcare providers to verify adherence remotely. VDOT has been shown to offer greater convenience, flexibility, privacy, autonomy, and cost-effectiveness while maintaining comparable adherence outcomes^14^. However, some patients miss video submission mainly due to technology-related factors and personal factors such as TB-related stigma^12,15^.

TB-related stigma is a potential yet understudied barrier to VDOT adherence. Since VDOT requires patients to record videos of themselves taking medication, concerns about privacy, confidentiality, and stigma may lead to non-adherence^12,13^. TB-related stigma can be categorized into three key domains: self-stigma, public stigma, and anticipated stigma. Self-stigma occurs when individuals internalize negative societal perceptions, leading to shame, denial of symptoms, and reluctance to seek treatment. This internalization can result in social withdrawal and rejection of essential support systems, further compromising adherence^16–18^. Public stigma refers to the negative attitudes, stereotypes, and discriminatory behaviors directed toward individuals with TB, leading to social exclusion and reduced opportunities. Anticipated stigma is the fear of discrimination or negative social consequences if one’s TB status is disclosed, even in the absence of direct stigma^19^. This fear can deter individuals from engaging with healthcare services, including adherence-support interventions like VDOT.

Despite the growing adoption of VDOT in TB programs globally, few studies have explored the effect of stigma on patient engagement, retention, and adherence to video submission. This study aimed to assess the relationship between baseline TB-related stigma and missed VDOT submissions among patients enrolled in the DOT Selfie randomized controlled trial in Kampala, Uganda. Findings may inform the adaptation of VDOT while minimizing stigma in Uganda and other high-burden TB settings.

## Methods and materials

### Study design

This study was a secondary analysis of data from the DOT Selfie RCT, conducted between July 2020 and October 2021 in Kampala, Uganda^20^. The original trial aimed to evaluate the effectiveness of video directly observed therapy (VDOT), combined with behavioral incentives, in improving TB medication adherence compared to the standard directly observed therapy (DOT) in an urban African setting.

The current analysis focuses on assessing the effect of baseline TB-related stigma on non-adherence to VDOT, as measured by the number of missed VDOT video submissions over the six-month treatment period.

### Study setting

The DOT Selfie RCT parent study was conducted in five TB clinics across healthcare facilities in Kampala, Uganda: Lubaga PNFP Hospital, Mulago National Referral Hospital, Kawaala HCIV, Kitebi HCIV, and Kisenyi HCIV. These facilities serve as key centers for TB diagnosis, treatment, and adherence support in Kampala.

Kampala, Uganda’s capital, has a high TB burden, with an annual incidence of 201 cases per 100,000 population^21^. Treatment adherence remains a significant challenge, with success rates ranging from 53% to 77%, influenced by factors such as urban mobility, socioeconomic constraints, and limited healthcare accessibility^22^. The selected facilities represented a mix of public and private-not-for-profit (PNFP) health centers, ensuring a diverse patient population.

### Study population

The study included all 71 patients with TB assigned to the VDOT arm of the DOT Selfie RCT. Eligibility was based on the inclusion and exclusion criteria of the parent study, and the details of the methods are published elsewhere^20^.

### Inclusion criteria

During the DOT Selfie study, participants were required to have a confirmed TB diagnosis, be within the first month of treatment, be aged 18 to 65 years, plan to reside in Kampala throughout their treatment, provide signed informed consent, and be proficient in Luganda or English.

### Exclusion criteria

Patients with TB were excluded if they had confirmed multidrug-resistant (MDR) or extensively drug-resistant (XDR) TB, were too ill to participate in study procedures lasting up to two hours, had cognitive, visual, or motor impairments preventing video recording, lacked access to electricity to charge a smartphone, or lived in areas without cellular network coverage.

### Data collection

A trained research assistant conducted baseline interviews using a structured, interviewer-administered questionnaire, developed based on a review of existing literature. To ensure linguistic accuracy, the questionnaire was translated into Luganda and then back-translated into English. Information on participants’ TB diagnosis, sociodemographic characteristics, phone ownership, familiarity with smartphones and technology, transportation access, social and family support, privacy concerns, TB-related knowledge, and community perceptions of TB was collected.

The cumulative missed video submissions for each VDOT user were tracked through the HIPAA-compliant VDOT system, a secure cloud-based platform where submitted videos were stored. This tracking was performed by a trained study nurse.

### Key variables and definitions

The dependent variable, missed videos, is a count variable that represents the total number of TB medication adherence videos a participant failed to record and/or submit to the VDOT monitoring system over the study period.

The primary exposure is baseline TB-related stigma, conceptualized into overall stigma, self-stigma, anticipated stigma, and public stigma. Covariates include sex, age, education level, religion, marital status, employment status, average monthly income, household size, alcohol consumption, and HIV status.

To measure TB severity, we used a modified Bandim TB score. This score was based on symptoms the patient reported, such as cough, coughing up blood, difficulty breathing, chest pain, night sweats, and fever. We also included chest X-ray results (normal/abnormal) and body weight (BMI). Each symptom counted as one point. For BMI, patients got two points if it was below 16, one point if it was between 16 and 18.49, and 0 points if it was 18.5 or higher. The total score ranged from 0 to 8. Based on the total, TB severity was grouped as: Mild (score 0–3), Moderate (score 4–5), and Severe (score 6–8).

### Measurement of stigma

TB-related stigma was assessed using a 13-item excerpt adapted from the TB-Stigma Measurement Guidance tool developed by the USAID-funded, KNCV-led Challenge TB project.

Responses for four items were initially collected on a binary scale (Yes = 1, No = 0), where “Yes” indicated stigma and “No” indicated no stigma.

For seven additional items, responses were collected on a 4-point Likert scale (1 = “Strongly Agree,” 2 = “Agree,” 3 = “Disagree,” 4 = “Strongly Disagree”). These responses were later dichotomized, with “Agree” and “Strongly Agree” grouped as “Yes” (indicating stigma) and “Disagree” and “Strongly Disagree” grouped as “No” (indicating no stigma).

The remaining two items were also dichotomized. For the item assessing community perception of TB diagnosis, responses of “Ashamed/Embarrassed” and “Worried about being rejected” were grouped as “Ashamed” (stigma), while “Indifferent” was categorized as “Not ashamed” (no stigma). For the item assessing family response to TB diagnosis, responses indicating continued support (e.g., “Relationship will be the same,” “They will show more support and care”) were categorized as “Support” (no stigma). In contrast, responses indicating distancing or lack of support (e.g., “They will not show me support and care,” “They will keep their distance from me and no longer show support and care”) were classified as “No support” (stigma). All responses indicative of stigma were coded Yes = 1 and No = 0

A composite overall stigma score was created by summing responses across 13 items, with a maximum possible score of 13. Stigma levels were classified as low (less than or equal to the median stigma score, which was 9) and high (greater than 9).

Three stigma domains, self-stigma, anticipated stigma, and public stigma, were also constructed. Self-stigma and anticipated stigma were each assessed using three items, while public stigma was measured using seven items. Participants who answered “Yes” to at least one item in a domain were classified as experiencing that form of stigma (coded as 1 = Yes), while those who responded “No” to all items were classified as not experiencing it (coded as 0 = No).

### Data analysis

Descriptive statistics were used to summarize participants’ characteristics. Categorical variables, such as sex, education level, marital status, employment status, alcohol consumption, and HIV status, were presented as frequencies and percentages. Continuous variables, including age and the number of missed videos, were summarized using means, standard deviations, medians, and interquartile ranges (IQR), depending on the distribution. Given that the outcome variable (missed videos) was not normally distributed, the Mann-Whitney U test was used to compare the median number of missed videos across stigma categories (overall stigma, self-stigma, anticipated stigma, and public stigma). Statistical significance was determined at p ≤ 0.05.

For multivariable analysis, Poisson, negative binomial, or zero-inflated regression models were considered, given that the outcome variable (missed videos) was a count variable and exhibited right skewness. However, the variance (SD^2^ = 294) was substantially larger than the mean (mean = 15), indicating overdispersion and thus violating the equi-dispersion assumption of Poisson regression. A Negative Binomial regression model was selected as the most appropriate model to account for count data that exhibits overdispersion. This model provides more accurate standard errors and confidence intervals. Four separate models were fitted to examine the effects of overall TB-related stigma, self-stigma, anticipated stigma, and public stigma on missed videos while adjusting for sex, age, education, marital status, employment, household size, alcohol consumption, and HIV status. Adjusted incidence rate ratios (IRRs) with 95% confidence intervals (CIs) were reported.

During stepwise model building, the Wald test assessed the contribution of each covariate, with a significant chi-square test (p ≤ 0.05) indicating a meaningful effect. Multicollinearity was checked using Pearson’s correlation, and variables with r ≥ 0.4 were excluded. Model fit was evaluated using AIC and BIC, with lower values indicating better performance. All analyses were conducted using STATA 14.2 (Stata Corp LLC, College Station, Texas). Results were presented using tables and box-and-whisker plots for clarity and visualization.

## Results

### Background characteristics of VDOT users

The study included 71 participants with a mean age of 33 years (SD = 12). The sample was nearly evenly distributed by sex (49% male, 51% female). Regarding the highest level of education, 38% had no formal education or primary education. More than half (59%) were employed. Comorbidities were present in 42% of participants, and 32% were HIV-positive (Table 1).

### Prevalence of baseline TB-related stigma among VDOT users

#### Overall TB-related stigma

TB-related stigma was prevalent among VDOT users, with 51% (95% CI: 39–62) experiencing high overall stigma.

#### Self-Stigma

The composite prevalence of self-stigma was 80% (95% CI: 22–45). While a majority of participants (82%) reported no fear or shame in disclosing their TB diagnosis to family or household members, 47% expressed feelings of shame or concern if the community became aware. Furthermore, 78% reported discomfort taking TB medication in the presence of community members.

#### Anticipated Stigma

The composite prevalence of anticipated stigma was 68% (95% CI: 55–78). Most participants (82%) did not find it difficult to seek support from family or household members, and 90% believed their families would continue to support them. However, 62% felt that community members would be unlikely to offer support, and 14% were unsure.

#### Public Stigma

Public stigma was highly prevalent, with a composite prevalence of 97% (95% CI: 90–100). A large proportion of respondents believed that TB is associated with social exclusion: 89% agreed that some individuals may not want to eat or drink with friends who have TB, 92% noted that people tend to keep their distance from patients with TB, 79% believed that even family members with TB may be avoided during meals, and 71.8% felt that community members may not want patients with TB living among them (Table 2).

#### Bivariate analysis of TB-related stigma and the number of missed VDOT

Among patients with TB using VDOT, those experiencing high overall stigma had a higher median number of missed video submissions (14; IQR: 3–29) compared to those with low stigma (5; IQR: 2–21), although this difference was not statistically significant (p = 0.3472). Similarly, patients reporting self-stigma missed more videos (median: 7; IQR: 2–24) than those without self-stigma (median: 3; IQR: 3–11), but the difference was also not significant (p = 0.2644).

**Figure 1:**
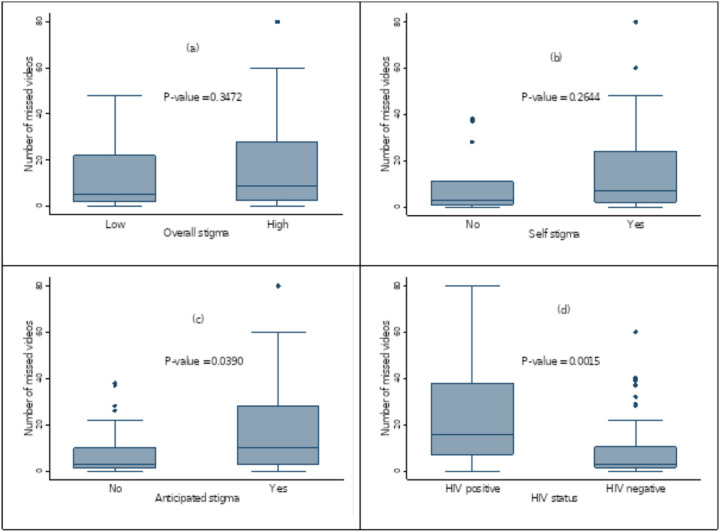
Box and whisker plots showing median number of missed videos among VDOT users

In contrast, anticipated stigma showed a statistically significant association, with participants who reported anticipated stigma missing more videos (median: 10; IQR: 3–28) than those without (median: 3; IQR: 1–10; p = 0.0390). Furthermore, patients co-infected with TB and HIV had a significantly higher number of missed video submissions (median: 16; IQR: 7–38) compared to those who were HIV-negative (median: 3; IQR: 2–11; p = 0.0015) (Fig. 1).

#### Association between TB-related stigma and the number of missed videos among VDOT users

Overall and anticipated TB-related stigma were significantly associated with the number of missed VDOT videos. Specifically, high levels of overall stigma were associated with a 90% increase in the rate of missed videos compared to low stigma levels (IRR: 1.9; 95% CI: 1.1–3.5), controlling for alcohol consumption and TB severity. Similarly, patients who reported anticipated stigma at baseline missed twice as many VDOT videos as those without anticipated stigma (IRR: 2.1; 95% CI: 1.2–3.8), controlling for marital status, household size, and HIV status.

In contrast, self-stigma and public stigma were not significantly associated with the number of missed VDOT videos (Table 3).

## Discussion

This study aimed to assess whether baseline TB-related stigma predicts missed doses during treatment using VDOT. Notably, a high proportion of participants (32%) reported high levels of overall stigma, signaling the enduring challenge of stigma in TB care. This high prevalence aligns with findings from studies conducted in other high TB-burden settings. For instance, a nationwide study in Ethiopia found that 38% of patients with TB experienced high levels of stigma^23^. Similarly, research conducted in an urban slum in Kampala found that over 50% of the patients with TB experienced a high level of TB stigma^24^. Our study shows that overall stigma and specifically anticipated stigma negatively affect video submission, thus significantly impacting engagement and adherence with VDOT use.

To our knowledge, this is the first study to evaluate the effect of stigma on the use of VDOT in Africa. When disaggregated by domain, public stigma emerged as the most prevalent form of TB-related stigma among participants, followed by self-stigma and anticipated stigma. This pattern is similar to what was found in a study in urban Zambia, indicating that people in the community often showed strong negative attitudes toward patients with TB, such as avoiding them or treating them unfairly^25^. This public stigma was more common than the shame patients felt about having TB (self-stigma) or their fear of how others might react (anticipated stigma)^25^. Similar patterns were reported in India, where stigma was deeply rooted in community misconceptions about TB transmission, its association with HIV, and broader social taboos^26^. Because of these beliefs, people with TB often expect others to treat them badly and may start feeling ashamed of themselves, leading to either anticipated or self-stigma.

A key finding from this study is the significant impact of anticipated stigma, which is defined as the fear of being judged or treated differently if one’s TB-positive status is known. Although VDOT is designed to be discrete and supportive, participants with high anticipated stigma missed more video submissions. This suggests that fear of disclosure can disrupt treatment, particularly in settings where privacy is limited. Similar findings have been reported in studies from South Africa and Thailand, where anticipated stigma was linked to poor adherence and delayed care-seeking^27–29^. These results highlight that perceived social judgment can be as harmful as actual discrimination; therefore, addressing it is critical for improving adherence and engagement with VDOT and other digital adherence technologies in TB care.

In addition to overall stigma and anticipated stigma, HIV-positive status was also significantly associated with increased missed VDOT submissions across all three models. This may be attributed to the compounded burden of managing two chronic, highly stigmatized illnesses, TB and HIV, which often involve complex treatment regimens, pill burden, and fears of discrimination. Patients co-infected with TB and HIV are likely to experience Syndemic stigma, wherein the interaction of HIV-related stigma and TB-related stigma amplifies barriers to treatment adherence. This dual stigma has been shown to negatively influence health-seeking behaviors and adherence in similar settings^30^. The amplified stigma may therefore contribute to the higher rates of missed VDOT videos observed among patients with HIV.

Larger household size was significantly predictive of fewer missed VDOT submissions, suggesting that living with more people may amplify social support and attenuate the effect of stigma during treatment. In such households, family members may offer practical help, reminders, and emotional encouragement, which factors promote adherence. This observation is consistent with studies from Uganda and Ethiopia, which have highlighted the protective influence of family and community networks in supporting TB treatment engagement^31,32^.

In contrast, the finding that married individuals missed more doses seems counterintuitive, as marriage is often assumed to provide the needed social support. However, some studies have shown that underlying complexities in relationships may lead to fear of disclosure of disease status to a partner, leading the interference with consistent medication intake^33^. A study done in Ghana highlighted that women felt more comfortable disclosing their disease status to other family members, but not their partners^34^. Future studies should explore how specific household structures, such as multigenerational living arrangements, marital dynamics, or household roles, impact VDOT adherence. Understanding these dynamics may help tailor adherence interventions to the social realities of patients’ living environments.

This study has two major limitations. First, stigma was assessed only at baseline; therefore, it does not account for any changes that may have occurred over time. It is possible that stigma could have reduced in response to VDOT use and increased social support, potentially underestimating its evolving influence on treatment monitoring adherence. Second, the study did not measure the mental health status or functional capacity of participants, both of which may have influenced adherence behaviors, as conditions such as depression, anxiety, or physical impairment are known to affect treatment engagement and consistency. Nonetheless, a notable strength of this study is its innovative integration of domain-specific stigma measures with real-time digital adherence data. By linking baseline stigma, particularly anticipated stigma, with VDOT video submissions, this analysis offers rare and timely empirical insight into how internalized fears can predict and undermine adherence, even within a discrete technology-enabled treatment model.

## Conclusions

This study demonstrates that TB-related stigma, especially the fear of judgment or rejection, negatively affects the use of VDOT. To harness the full potential of digital adherence technologies, TB programs must prioritize routine stigma screening and counseling from treatment initiation, alongside community campaigns to transform harmful public attitudes. Tailored interventions for TB-HIV coinfected individuals should be integrated into the care models. Further research should seek to evaluate how the changes in stigma over time impact the use of VDOT.

## Figures and Tables

**Table 1:** Background characteristics of VDOT users

Variables	Frequency (%)
**Sex**	
Male	35 (49)
Female	36 (51)
**Age, Mean (SD)**	33 (12)
**Age category**	
18 to 24 years	19 (27)
25 to 39 years	32 (45)
40 + years	20 (28)
**Highest level of education**	
No formal education or Primary	27 (38)
Secondary	28 (39)
Tertiary	16 (23)
**Religion**	
Catholic	19 (27)
Protestant	18 (25)
Muslim	17 (24)
Pentecostal and others	17 (24)
**Marital status**	
Never married	36 (51)
Currently married	22 (31)
Previously married	13 (18)
**Employment status**	
Employed	42 (59)
Unemployed	29 (41)
**Monthly income**	
Less than USD 27.4	33 (47)
USD 27.5–109.6	23 (32)
Greater than USD 109.6	15 (21)
**Household size, category**	
One member	12 (17)
Two to four members	30 (42)
More than four members	29 (41)
**Alcohol consumption**	
Yes	11 (16)
No	60 (84)
**Comorbidities excluding HIV/AIDS**	
Absent	41 (58)
*Present	30 (42)
**HIV status**	
Positive	23 (32)
Negative	48 (68)

[1 USD = 3,651.2 Uganda Shillings as of May 2025]

* Present means having Hypertension, Diabetes, Chronic Heart Disease (CHD), or Joint disease

**Table 2:** Responses to the 13 TB-related stigma items

Item	n (%)
**Self-stigma**	
Do you feel afraid or ashamed of telling any family or household members about your TB diagnosis?	
No	58 (82)
Yes	13 (18)
How would you feel if anyone in the community found out about your TB diagnosis?	
Not worried	38 (53)
Shame/Worry	33 (47)
Would you be comfortable taking your TB medicine in the presence of any person from your community?	
No	55 (78)
Yes	16 (22)
**Anticipated stigma**	
Do you think it would be difficult for you to ask your family or household members for the support and care you need because you have TB	
No	58 (82)
Yes	13 (18)
If your family or household members find out about your TB diagnosis, how do you think it will affect your relationship with them	
No support	7 (10)
Support	64 (90)
Do you think that people in your community would offer any needed support to you even if they know you have TB disease	
No	44 (62)
Yes	17 (24)
Don’t know	10 (14)
**Public stigma**	
Some people may not want to eat or drink with friends who have TB	
No	8 (11)
Yes	63 (89)
Some people feel uncomfortable about being near a person who has had TB	
No	8 (11)
Yes	63 (89)
Some people do not want those with TB playing with their children	
No	8 (11)
Yes	63 (89)
Some people keep their distance from people with TB	
No	6 (8)
Yes	65 (92)
Some people do not want to talk to others with TB	
No	16 (22)
Yes	55 (78)
Some people may not want to eat or drink with family members who have TB	
No	15 (21)
Yes	56 (79)
Prefer not to have people with TB living in their community	
No	20 (28)
Yes	51 (72)

**Table 3:** Multivariable analysis for the association between TB-related stigma and the number of missed videos

Variables	Model 1	Model 2	Model 3	Model 4^a^
**Stigma**				1.1 (0.8, 1.2)
No	1.0	1.0	1.0	
Yes	1.9 (1.1, 3.5) *	1.5 (0.7, 3.2)	2.1 (1.2, 3.8) *	
**Marital status**				
Never married	-	1.0	-	1.0
Currently married	-	2.4 (1.3, 4.6) **	-	2.3 (1.2, 4.5) *
Previously married	-	2.1 (1.1, 4.0) *	-	1.9 (1.0, 3.7) *
**Household size**		0.8 (0.7, 0.9) ***	0.9 (0.8, 0.9) **	0.8 (0.7, 0.9) ***
**Consumes alcohol**				
No	1.0	-	1.0	-
Yes	2.8 (1.5, 4.9) **	-	1.9 (1.2, 3.2) *	-
**TB severity**				
Mild	1.0	-	-	-
Moderate	1.6 (0.9, 3.1)	-	-	-
Severe	2.0 (1.1, 3.9) *	-	-	-
**HIV status**				
Negative	-	1.0	1.0	1.0
Positive	-	2.2 (1.2, 3.8) **	2.0 (1.2, 3.4) **	2.3 (1.4, 3.9) **

* P ≤ 0.05,

** P < 0.01,

*** P < 0.001

**Model 1**: Overall stigma, **Model 2**: Self-stigma, **Model 3**: Anticipated stigma, and **Model 4**: Public stigma

## Data Availability

De-identified data are available upon request, subject to approval and data use agreements with the University of Georgia and Makerere University.

## Abbreviations

DOT: Directly Observed Therapy
VDOT: Video Directly Observed Therapy
TB: Tuberculosis
DATs: Digital Adherence Technologies
MDR-TB: Multi-Drug Resistant Tuberculosis
XDR-TB: Extremely Drug-Resistant Tuberculosis
HIV: Human Immunodeficiency Virus
HIPAA: Health Insurance Portability and Accountability Act
NIH: National Institutes of Health
